# Mixture SNPs effect on phenotype in genome-wide association studies

**DOI:** 10.1186/1471-2164-16-3

**Published:** 2015-02-03

**Authors:** Ling Wang, Haipeng Shen, Hexuan Liu, Guang Guo

**Affiliations:** Department of Statistics and Operation Research, University of North Carolina-Chapel Hill, 27514 Chapel Hill, USA; Department of Sociology, University of North Carolina-Chapel Hill, 27514 Chapel Hill, USA; Innovation and Information Management, School of Business, Faculty of Business and Economics, University of Hong Kong, Hong Kong, PR China; Carolina Center for Genome Sciences, University of North Carolina-Chapel Hill, 27514 Chapel Hill, USA; Caroline Population Center, University of North Carolina-Chapel Hill, 27514 Chapel Hill, USA

**Keywords:** Bayesian variable selection, Genome-wide association studies, Gibbs sampling

## Abstract

**Background:**

Recently mixed linear models are used to address the issue of “missing" heritability in traditional Genome-wide association studies (GWAS). The models assume that all single-nucleotide polymorphisms (SNPs) are associated with the phenotypes of interest. However, it is more common that only a small proportion of SNPs have significant effects on the phenotypes, while most SNPs have no or very small effects. To incorporate this feature, we propose an efficient Hierarchical Bayesian Model (HBM) that extends the existing mixed models to enforce automatic selection of significant SNPs. The HBM models the SNP effects using a mixture distribution of a point mass at zero and a normal distribution, where the point mass corresponds to those non-associative SNPs.

**Results:**

We estimate the HBM using Gibbs sampling. The estimation performance of our method is first demonstrated through two simulation studies. We make the simulation setups realistic by using parameters fitted on the Framingham Heart Study (FHS) data. The simulation studies show that our method can accurately estimate the proportion of SNPs associated with the simulated phenotype and identify these SNPs, as well as adapt to certain model mis-specification than the standard mixed models. In addition, we analyze data from the FHS and the Health and Retirement Study (HRS) to study the association between Body Mass Index (BMI) and SNPs on Chromosome 16, and replicate the identified genetic associations. The analysis of the FHS data identifies 0.3*%* SNPs on Chromosome 16 that affect BMI, including rs9939609 and rs9939973 on the *FTO* gene. These two SNPs are in strong linkage disequilibrium with rs1558902 (Rsq =0.901 for rs9939609 and Rsq =0.905 for rs9939973), which has been reported to be linked with obesity in previous GWAS. We then replicate the findings using the HRS data: the analysis finds 0.4*%* of SNPs associated with BMI on Chromosome 16. Furthermore, around 25*%* of the genes that are identified to be associated with BMI are common between the two studies.

**Conclusions:**

The results demonstrate that the HBM and the associated estimation algorithm offer a powerful tool for identifying significant genetic associations with phenotypes of interest, among a large number of SNPs that are common in modern genetics studies.

## Background

Genome-wide association studies (GWAS) have successfully identified genetic loci association with complex diseases and other traits. SNPs identified by traditional GWAS can only explain a small fraction of the heritability, due to the strict multiple-comparison significance requirement when testing each SNP individually. For example, Vissher [1] discussed 54 loci associated with height which only explained 5*%* heritability; [2] described 32 loci associated with Body Mass Index (BMI) which explained 1.45*%* of the variance in BMI. More recently, [3] used mixed linear models (MLM) to simultaneously take into account all the SNPs, which is shown to alleviate the missing-heritability issue.

In this study, we extend the work of [3] to identify the subset of SNPs that are significantly associated with the phenotype of interest, instead of assuming all the SNPs are associative, through a Hierarchical Bayesian model (HBM). Similar to [3], all SNPs are considered simultaneously to estimate the heritability, instead of one by one as in the traditional GWAS, hence our HBM also helps to capture missing heritability. Different from the authors in [3], we assume that the SNP effects are distributed as the mixture of a point mass at zero, for those non-effective SNPs, 6 and a normal distribution for those associative SNPs.

Our proposed Hierarchical Bayesian model (HBM) can be represented using the following set of Eqs. 1 and (2). Eq. 1 follows the same set-up as [3]: **Y** is the *n*×1 response vector which corresponds to the individuals’ phenotype in our study, **X** is the design matrix for the fixed effects, **W** is the standardized genotype matrix, and the vector *b* contains the *N* SNP random effects, where the *j*th element is the random effect corresponding to the *j*th SNP and is assumed to follow the mixture distribution as in (2), depending on the latent indicator *I*_*j*_, *j*=1,…,*N*:
1

where
2

One key contribution of our HBM is its capability of automatically selecting significant SNPs while simultaneously incorporating all the SNPs. Eq. 2 is the technical reason behind the selection feature, which can be intuitively understood as follows. Imagine that each SNP is coupled with one Bernoulli indicator *I*_*j*_ with success probability *p*, and all the *N* Bernoulli indicators are independent. The SNPs then fall into two categories, where the first category contains those with *I*_*j*_=1, which are the 100×*p%* associative SNPs with effects following a normal distribution, while the second group includes the remaining SNPs with *I*_*j*_=0, who have zero effects. The selection of the associative SNPs is achieved through identifying the SNPs with *I*_*j*_=1, which are chosen to be those with the largest posterior probability of being 1, through the HBM algorithm as described below:


Several Bayesian variable selection algorithms have been proposed through hierarchical modeling, with applications in genomic studies [4]. Considered a variational Bayes algorithm for GWAS. This method approximates the joint posterior density of the hierarchical regression model with a factorized form and minimizes the Kullback-Liebler distance between the factorized form and the full posterior distribution. Although this method is fast to compute, the accuracy of prediction depends on how well the factorized form approximates the posterior distribution of the hierarchical model [5]. Developed a Bayesian variable selection regression algorithm to solve the hierarchical model. They adopted several strategies to improve computational performance, for example, they used marginal associations of the SNPs on the traits as the initial screen step for the latent indicator *I*_*j*_ in (2) [6]. This indicates that the distribution of the random effect *b*_*j*_ is similar to the marginal estimates of the SNP effects on the traits.

In this study, we modify the standard MCMC algorithm based on the stochastic search algorithm proposed by [7]. The algorithm directly samples the parameters from their posterior distributions and obtain the inferences for the parameters. Because the number of SNPs is large, each iteration of the algorithm involves matrix inversion with the dimension being the number of SNPs. To reduce computation time, we modify the algorithm by sampling the random effects *b*_*j*_ conditional on the indicator *I*_*j*_. The modified algorithm significantly reduces computation time, especially when the number of SNPs is large and the mixture probability *p* is small, while is still able to identify the significant predictors accurately. Detailed description of the algorithm will be stated in Section “Methods”: Method. We also implement several computing tricks so that the algorithm can be used to estimate models with the number of SNPs in the order of 100,000 (Section “Example 2”).

Our HBM is first applied to analyze simulated data sets in Section “Simulation studies” to show that the proposed algorithm is able to identify the SNPs that are significantly associated with the phenotype and correctly estimate the model parameters as well as PVE, which is defined as the proportion of total genetic variance over total phenotypic variance:
3

where  is the total genetic variance which equals  in (2) times the number of SNPs. The total phenotypic variance is the sum of the genetic variance  and the variance of the error terms of *ε* in (2), denoted as .

We also compare HBM with the Genome-wide Complex Trait Analysis (GCTA) proposed by [3]. The basic concept of GCTA is to fit the effects of all the SNPs as random effects using a mixed linear model (MLM). Note that the MLM is a special case of our HBM when *p*=1. It is shown in our studies that if a large number of SNPs have small/noisy effects on the phenotype, the MLM tends to over-estimate the PVE while the HBM is still able to correctly estimate it. We present in Section “Real data set results” two real data applications through the Framingham Heart Study [8] and the Health and Retirement Study [9], where we study the association between the SNPs on Chromosome 16 and the phenotype body mass index (BMI). We are able to identify associative SNPs on the *FTO* gene which are consistent with earlier findings in the literature and replicate the results in the two studies.

## Results and discussion

### Simulation studies

The performance of the HBM and MLM is illustrated using two simulated examples with the identical simulation settings but different number of random effects. Example 1 (Section “Example 1”) considers 10,000 random effects, while Example 2 (Section “Example 2”) has 100,000 random effects and is closer to the scale of real GWAS. Each example also consists of two simulation cases: in Case 1 the random effects follow a mixture distribution of a point mass at zero and a normal distribution, while in Case 2, the random effects follow a mixture of two normal distribution with one of the two has a very small variance, trying to mimic scenarios with a large number of small/noisy effects on the phenotype.

For both simulated examples, genotype information of the individuals from the Framingham Heart Study (FHS) is used as input matrix. Detailed description of the FHS data is provided in Section “The Framingham heart study”.

#### Example 1

In this example, we randomly select 10,000 SNPs on Chromosome 16 of the FHS data and use them as the input genotype matrix, **W**. The trait **Y** is then simulated according to the following model:
4

where **W** is the standardized genotype matrix and *b* is the allelic effect of the SNPs that will be simulated. The residual effect (*ε*) is generated from a normal distribution with a mean of zero and variance of . As discussed above, two simulation cases are generated as follows.

- **Simulation Case 1:** The random effect *b* follows a mixture distribution of a point mass at zero plus a normal distribution. In this situation, the SNPs are either associated with the phenotype (whose random effects are distributed as a normal distribution) or not associated with the phenotype (whose random effects will be zero);
- **Simulation Case 2:** The random effect *b* follows a mixture of two normal distributions with one of the two distributions has a very small variance. In practice, many SNPs might have very small/noisy effects on the complex traits [10]; hence, we are simulating those scenarios with letting some of the SNPs have noisy effects on the phenotype that are normally distributed with a very small variance.

For Simulation Case 1, we randomly select 100×*p%* of the SNPs as the ones associated with the phenotype (namely, the association SNPs), and draw their random effects *b* from the distribution , and treat the remaining SNPs as non-association SNPs with zero effects. We then fix the PVE at the predetermined value, and simulate the residual *ε* from the distribution  where . Phenotype *y* is generated using *W*, *b* and *ε* according to Eq. 4. For Simulation Case 2, the data set is generated in a similar way as in Case 1, with the only difference being that the random effects for the non-association SNPs are simulated from  where *σ* is a very small number (e.g. *σ*=0.01) instead of zero.

Table 1 shows the estimation results from the simulated data sets using the HBM and MLM along with the true model parameters. The estimated mixture probability  and the random effect variance  by the HBM are close to their corresponding true values, 0.01 and 0.1, respectively. This demonstrates the good performance of our estimation method. In both simulation cases, the MLM severely underestimates , as it divides the total genetic variance onto all the SNPs, instead of just the 1% association SNPs (*p*=0.01), which results in underestimation of the genetic effects. In addition, in Simulation Case 2, the estimated PVE from the MLM is much larger than the true value while the HBM gives a closer PVE estimate. The reason is that the MLM can not distinguish the “significant” SNP effects versus those “noisy” effects due to its assumption that all random effects follow the same distribution. Therefore,  obtained by MLM would include both “significant” and “noisy” effects and thus lead to overestimation of PVE according to (3). We comment that the simulation model in this case is different from the underlying models assumed by our HBM and the MLM of GCTA. As the results indicate, the HBM is rather robust against such model misspecification.

**Table 1:** **Example 1 - estimation results**

		Simulation Case 1			Simulation Case 2	
Parameters						
	True value	Estimates from	Estimates from	True value	Estimates from	Estimates from
		HBM (s.e) ^***a***^	MLM (s.e) ^***a***^		HBM (s.e) ^***a***^	MLM (s.e) ^***a***^
	0	0.01 (0.06)	0.03 (0.06)	0	0.02 (0.02)	0.02 (0.02)
	0.1	0.11 (0.06)	0.011 (0.006)	0.1	0.11 (0.05)	0.012 (0.004)
	1	0.94 (0.24)	1.12(0.04)	1	1.03 (0.03)	1.04 (0.05)
	1	1.21 (0.13)	1.19 (0.20)	1	1.13 (0.35)	1.62 (0.65)
	0.01	0.007 (0.003)	-	0.01	0.008 (0.002)	-
PVE ^*c*^	0.5	0.53 (0.12)	0.52 (0.21)	0.5	0.52 (0.15)	0.61 (0.13) ^*b*^
Number of Random Effects		10,000				

^*a*^Values in parenthesis are standard errors. ^*b*^Genetic Variance _*g*_^2^ is defined in the same way in [11] which equals to _*b*_^2^ ×*N*. N is the number of SNPs whose effect *b*_*j*_ follows the (0, *σ*_*b*_^2^) distribution. ^*c*^PVE is calculated as .

#### Example 2

This simulation example is used to demonstrate the performance of the HBM algorithm when the number of SNPs is large (i.e. 100,000), in the order of real GWAS. We have to implement several computational optimizing strategies in order to speed up the computation on such a large number of SNPs as well as to efficiently use the computer memory.

First, in each iteration of the HBM algorithm, we need to invert a square matrix with the rank the same as the number of SNPs. Instead of inverting this matrix directly, we employ the Sherman-Morrison-Woodbury formula [12], to change the matrix inversion to one that only has the same rank as the number of observations, which usually is much smaller than the number of SNPs in genetic studies. Secondly, computation using a large number of SNPs is intensive. Analyzing large datasets of SNPs seems to be impractical on uniprocessor machines. Thus, we carry out the analysis in parallel on UNC-CH’s multi-core Linux-based cluster computing server. We write scripts to distribute the computation among multiple cores/CPUs and run multiple computing analyses simultaneously. Our study shows that parallel computing can speed up the computation by a factor of 20 on a 10-core computing node on the cluster. It takes 668.5 minutes and 158GB memory to finish the calculation for the simulated data set with 100,000 SNPs. To consider whole genome data with even more SNPs, the amount of memory and computation power of the server will be the main bottleneck.

Similar to Example 1 in Section “Example 1”, we consider the same two simulation cases. The estimation results are summarized in Table 2. Even for the larger number of SNPs, our HBM still performs well in both cases, while the same drawbacks exist for the MLM.

**Table 2:** **Example 2 - estimation results**

		Simulation Case 1			Simulation Case 2	
Parameters						
	True value	Estimates from	Estimates from	True value	Estimates from	Estimates from
		HBM (s.e) ^***a***^	MLM (s.e) ^***a***^		HBM (s.e) ^***a***^	MLM (s.e) ^***a***^
	0	0.12 (0.08)	0.12 (0.13)	0	0.09 (0.09)	0.12 (0.07)
	0.1	0.13 (0.01)	0.005 (0.002)	0.1	0.13 (0.05)	0.009 (0.002)
	3	2.86 (1.05)	3.15 (1.45)	3	3.67 (1.13)	3.18 (1.05)
	3	2.37 (1.34)	2.53 (2.01)	3	3.17 (2.13)	8.1 (3.25)
	0.003	0.0024 (0.001)	-	0.003	0.0025 (0.001)	-
PVE	0.5	0.45 (0.24)	0.44 (0.23)	0.5	0.46 (0.25)	0.72 (0.33) ^*b*^
Number of Random Effects		100,000				

^*a*^Values in the parenthesis are standard errors. ^*b*^The PVE estimated by MLM is higher than the true values in both simulation cases.

For Example 2 (with 100,000 SNPs), Table 3 reports the cross table results of the association SNPs identified by HBM against the truth. As one can see, the HBM can correctly detect 76*%* and 82*%* of the true association SNPs for the two simulation cases respectively, and more than 99.9% of the true non-association SNPs. This suggests that the HBM works very well at detecting association SNPs with the false positive rate as low as 0.062*%* and 0.041*%*, respectively.

**Table 3:** **Example 2 - detection results from HBM**

	Simulation Case 1		Simulation Case 2	
	Association SNPs	Non-association SNPs	Association SNPs	Non-association SNPs
	identified by HBM	identified by HBM	identified by HBM	identified by HBM
Association SNPs	76*%*	24*%*	82*%*	18*%*
Non-Association SNPs	0.062*%*	99.938*%*	0.041*%*	99.959*%*

### Real data set results

#### The Framingham heart study

We further apply HBM and MLM to data from the Framingham Heart Study [8] to study genetic associations with the body mass index (BMI). The FHS is a community-based, prospective, longitudinal study following three generations of participants.

Genotyping for FHS participants was performed using the Affymetrix 500K GeneChip array. Genotypes on the Y chromosome are not included in our analysis. A standard quality control filter is applied to the genotype data. Individuals with 5*%* or more missing genotype data were excluded from analysis. SNPs that are on the X chromosomes and have a call rate ≤99*%* or a minor allele frequency ≤0.01 were also eliminated from the analysis. The application of the quality control procedures resulted in 8,738 individuals with 287,525 SNPs from the 500,000 genotype data. Genotype data were converted to minor allele frequencies for the analysis. One individual of a pair is deleted if the genetic relationship is greater than 0.025. Note that the genetic relationship between individual *j* and individual *k* is defined as in [3]:
5

where *x*_*ij*_/*x*_*ik*_ is the number of copies of the reference allele for the *i*^*th*^ SNP of the *j*^*th*^/*k*^*th*^ individual and *p*_*i*_ is the frequency of the reference allele. After the above preprocessing, there are 1,915 unrelated individuals in the analysis.

Because the total number of SNPs in the FHS data is close to 300,000, computation is limited by the memory of the UNC server if we include all SNPs in the analysis. Therefore, as a proof of concept, the 13,764 SNPs on Chromosome 16 are used in the analysis. Another reason for considering this chromosome is that it contains an enzyme fat mass and obesity associated protein also known as *FTO*. We would like to see whether the HBM can identify the SNPs that are significantly correlated with BMI on Chromosome 16, especially those SNPs on the *FTO* gene. We include the first seven Principle Components (PCs) for BMI as fixed effects in the model to eliminate genotype correlation induced by biological ancestry.

The estimation results are shown in the left panel of Table 4. We see that the estimated mixture probability  from HBM is around 0.3*%*, which indicates only 0.3 percent of the SNPs on Chromosome 16 are associated with BMI.

**Table 4:** **Real data estimation results using HBM and MLM**

	The Framingham heart study ^***a***^		The health and retirement study ^***b***^	
	Estimates from	Estimates from	Estimates from	Estimates from
Parameter	HBM (s.e) ^***b***^	MLM (s.e) ^***b***^	HBM (s.e) ^***b***^	MLM (s.e) ^***b***^
(Intercept)	26.42 (0.29)	26.46 (0.28)	27.47 (0.07)	27.47 (0.05)
	0.20 (0.09)	0.014 (0.005)	0.014 (0.009)	0.0001 (0.00002)
	24.68 (0.38)	22.64 (0.41)	22.32 (0.23)	27.29 (0.41)
	1.49 (0.40)	3.49 (0.35)	0.6678 (0.4)	0.95 (0.38)
	0.0034 (0.0005) ^*c*^	-	0.0042 (0.0004) ^*c*^	-
PVE	0.06 (0.01) ^*d*^	0.13 (0.01) ^*d*^	0.026 (0.01)	0.04 (0.01)
*P* *C*1	-19.01 (44.72)	-10.90 (44.15)	82.75 (33.56)	91.66 (20.10)
*P* *C*2	-18.00 (24.41)	-18.27 (24.90)	-15.54 (8.52)	-18.41 (10.49)
*P* *C*3	2.63 (8.91)	5.65 (10.25)	26.01 (9.32)	23.01 (6.91)
*P* *C*4	-10.13 (9.11)	-10.05 (9.45)	3.52 (3.24)	2.24 (6.83)
*P* *C*5	18.55 (9.44)	19.80 (9.89)	14.43 (11.14)	8.49 (6.79)
*P* *C*6	-4.75 (12.17)	-4.49 (12.02)	-9.77 (14.34)	-19.81 (6.77)
*P* *C*7	16.06 (12.46)	13.09 (12.48)	-1.21 (11.95)	-2.39 (6.74)

^*a*^The analysis is based on 1,915 unrelated persons in the Framingham Heart data set using 13,764 SNPs on Chromosome 16 to predict BMI. ^*b*^The analysis is based on 12,237 unrelated persons in the Health and Retirement Study using 11,925 SNPs on Chromosome 16 to predict BMI. ^*c*^Values in the parenthesis are standard errors. ^*d*^PVE estimated by MLM is higher than that estimated by HBM.

The top panel of Table 5 lists the 43 SNPs that are identified by HBM as associated with BMI, which are ordered according to their names, along with the corresponding genes if available. Among these identified SNPs, the SNP rs9939609 variant has been found to be associated with obesity risk among children and adolescents of Beijing, China by [13], and with BMI and waist circumference among European- and African-American youth by [14]. The SNP rs9939973 on the *FTO* gene has also been found to be related with overweight of children in Korean by [15]. These two SNPs are in strong linkage disequilibrium (LD) with SNP rs1558902 (Rsq=0.901 for rs9939609 and Rsq=0.905 for rs9939973), which had been previously reported in a well-known GWAS [2]. The detection results can also be replicated to some extent: the three SNPs highlighted in red and the genes indicated in blue are also detected in the HRS analysis to be reported below in Section “The health and retirement study”.

**Table 5:** **Per allele change in BMI for association SNPs identified by HBM**

The Framingham heart study							
Gene^*a*^	SNPs^*b*^	Per allele change	Per allele change	Gene^*a*^	SNPs^*b*^	Per allele change	Per allele change
		in BMI by HBM	in BMI by MLM			in BMI by HBM	in BMI by MLM
CDH13	rs4508407	0.249	0.009	NAA60	rs12448488	0.209	0.013
CMIP	rs2966097	0.187	0.011	PRMT7	rs3785114	0.201	0.006
FTO	rs9939609	0.149	0.01	RABEP2	rs7184597	0.016	0.006
FTO	rs9939973	0.224	0.014	SDR42E1	rs11443	0.204	0.011
RBFOX1	rs11641750	0.225	0.012	SHISA9	rs149917	0.228	0.009
RBFOX1	rs17137899	0.245	0.01	WDR59	rs4888320	0.105	0.012
RBFOX1	rs17140501	0.039	0	ZNF423	rs4785325	0.214	0.014
SLC38A8	rs12716746	0.128	0.007		rs11860830	0.252	0.008
SLC38A8	rs4782578	0.167	0.009		rs12325385	0.201	0.009
WWOX	rs17711186	0.197	0.013		rs12447727	0.108	0.011
ATP2C2	rs962877	0.05	0.003		rs1318275	0.06	0
CACNG3	rs11648890	0.075	0.007		rs16947390	0.064	0.005
CENPN	rs1048194	0.082	0.002		rs17503512	0.004	0.001
CKLF-CMTM1	rs896086	0.099	0.01		rs2626640	0.148	0.007
KLHDC4	rs4843689	0.23	0.008		rs2631530	0.264	0.011
LOC101927676	rs1103775	0.17	0.009		rs30121	0.161	0.001
LOC101927998	rs328345	0.057	0.007		rs4784621	0.023	0.001
LOC102723396	rs4399544	0.078	0.008		rs7201071	0.109	0.009
MEFV	rs11466045	0.157	0.011		rs7202145	0.029	0.001
MGRN1	rs841224	0.094	0.008		rs8048671	0.239	0.018
MIR138-2	rs1529930	0.165	0.009		rs9921866	0.2	0.009
MKL2	rs4267326	0.264	0.008				
**The Health and Retirement Study**							
**Gene** ^***a***^	**SNPs** ^***b***^	**Per allele change**	**Per allele change**	**Gene** ^***a***^	**SNPs** ^***b***^	**Per allele change**	**Per allele change**
		**in BMI by HBM**	**in BMI by MLM**			**in BMI by HBM**	**in BMI by MLM**
CDH13	rs7199677	0.14	0.005	KIAA0513	rs8045387	0.112	0.001
CMIP	rs10514518	0.123	0.002	LOC102724927	rs2601773	0.112	0.002
FTO	rs9939609	0.143	0.003	MPHOSPH6	rs2303267	0.183	0.007
FTO	rs9940128	0.163	0.009	NDRG4	rs11076243	0.133	0.002
RBFOX1	rs11076998	0.162	0.004	PAPD5	rs7191151	0.129	0.003
RBFOX1	rs11647425	0.104	0.001	PSKH1	rs2136648	0.141	0.005
RBFOX1	rs12448747	0.173	0.004	RP11-488I20.3	rs13332284	0.202	0.011
RBFOX1	rs1473145	0.132	0.003	URAHP	rs9921920	0.121	0.008
RBFOX1	rs17562548	0.211	0.02	VAT1L	rs13330130	0.11	0.001
RBFOX1	rs1860304	0.174	0.006		rs11075417	0.147	0.003
SLC38A8	rs4782578	0.137	0.009		rs1362441	0.122	0.002
WWOX	rs16948787	0.111	0.004		rs154554	0.16	0.002
WWOX	rs4888855	0.223	0.019		rs16960867	0.151	0.005
BCAR1	rs4261573	0.118	0.001		rs4023915	0.155	0.006
CDH11	rs1520229	0.183	0.009		rs4467088	0.113	0.002
CLEC16A	rs767019	0.115	0.002		rs4784621	0.106	0.001
CMC2	rs2549855	0.111	0.002		rs7187990	0.104	0
CNGB1	rs7184838	0.172	0.012		rs8045580	0.126	0.005
CNTNAP4	rs4888514	0.178	0.008		rs964933	0.114	0.001
GPR139	rs868554	0.14	0.002		rs9925215	0.119	0.005

^*a*^The same genes are identified associated with BMI using both FHS and HRS data are shown in blue. ^*b*^SNPs identified to be associated with BMI in both FHS and HRS data are shown in red.

The predicted allele effects on BMI (*kg*/*m*^2^ per allele) by HBM and MLM are compared in Table 5, which are calculated as the posterior mean of the random effects under each model. The allele effect predicted by HBM is closer to the findings in the previous GWAS. As an example, we compare SNP rs9939973’s effect on BMI with rs1558902’s, both of which are on the *FTO* gene and are highly correlated [2], found that the per allele change in BMI for SNP rs1558902 is 0.39 (*kg*/*m*^2^) based on a total of 249,796 individuals of European ancestry using a GWAS method. It is much closer to the estimate obtained by HBM (0.224 *kg*/*m*^2^), rather than the much-lower estimate given by MLM (0.014 *kg*/*m*^2^). This comparison indicates that the MLM, assuming that every SNP has an effect on the phenotype, underestimates the SNP effects.

One can also see from Table 5 that the estimated PVEs are different (5.6*%* vs. 13.3*%*). In Section “Simulation studies”, we have shown that the MLM tends to overestimate PVE if there exist many SNPs with small/noisy effects on the phenotype, which we think is also the case for the FHS data here. To demonstrate that there exist SNPs with small effects on BMI, we perform the following multi-scale analysis by varying the amount of SNPs on Chromosome 16 to be included in MLM and showing how the corresponding estimated PVE changes. We first regress BMI on every single SNP and obtain the corresponding *p*-value. Then we consider a range of varying thresholds on the *p*-values, and only include those SNPs with a *p*-value below the threshold in the MLM when estimating the PVE. We systematically increase the *p*-value threshold so that more and more SNPs that are “less” significant will be included. The idea is that as the p-value threshold increases, more SNPs with small effects on BMI will be included when estimating PVE, which will result in higher PVEs. The estimation results are presented in Table 6. The estimated PVE decreases from 18*%* to 1*%* as a decreasing number of SNPs with smaller *p*-values (below the thresholds from 10^−1^ to 10^−7^) are included in the analysis. The results indicate that when estimating PVE using MLM, the more SNPs with small effects on BMI are included, the higher the estimated PVE is.

**Table 6:** **The Framingham Heart Study: PVE Estimation Using Proportion of SNPs Based on P-value Threshold** ^***a***^

	P-value <0 ***.*** 1 ^***bc***^	P-value <0 ***.*** 01 ^***bc***^	P-value <0 ***.*** 001 ^***bc***^	P-value <0 ***.*** 0001 ^***bc***^
	(s.e.)	(s.e.)	(s.e.)	(s.e.)
Number of SNPs	2690	561	145	45
Genetic Variance	4.45 (0.34)	3.34 (0.37)	2.08 (0.38)	0.86 (0.31)
Error Variance	20.66 (0.34)	22.31 (0.35)	24.06 (0.38)	25.25 (0.39)
Total Variance	25.11 (0.45)	25.65 (0.50)	26.14 (0.53)	26.11 (0.50)
PVE	0.18 ^*d*^ (0.06)	0.13 ^*d*^ (0.04)	0.08 ^*d*^ (0.03)	0.03 ^*d*^ (0.01)
	**P-value <0** ***.*** **00001** ^***bc***^	**P-value <0** ***.*** **000001** ^***bc***^	**P-value <0** ***.*** **0000001** ^***bc***^	
	**(s.e.)**	**(s.e.)**	**(s.e.)**	
Number of SNPs	21	10	7	
Genetic Variance	0.43 (0.21)	0.43 (0.28)	0.25 (0.22)	
Error Variance	25.48 (0.40)	25.60 (0.40)	25.73 (0.40)	
Total Variance	25.91 (0.45)	26.03 (0.49)	25.97 (0.46)	
PVE	0.02 ^*d*^ (0.01)	0.02 ^*d*^ (0.01)	0.01 ^*d*^ (0.01)	

^*a*^The analysis in the table is carried out using the GCTA software developed by [3]. ^*b*^P-value is obtained by regressing BMI on each single SNP. ^*c*^Values in the parenthesis are standard errors. ^*d*^PVE decreases from 18 *%* to 1 *%* as a smaller group of SNPs are included in the analysis.

In summary, the analysis of the Framingham data reveals several important empirical findings: (1) Among all the SNPs on Chromosome 16, only 0.3*%* of them are significantly associated with BMI according to HBM; (2) Several association SNPs identified by HBM have also been reported to be significantly related with BMI in previous studies; (3) The MLM tends to underestimate the allele effect on the phenotype while the HBM estimates much closer to previous GWA study results; (4) Because the MLM includes SNPs with small effects on BMI, the estimated PVE by MLM is much higher than the estimate from HBM.

#### The health and retirement study

In this section, we try to replicate the results in Section “The Framingham heart study” using data from the Health and Retirement Study [9]. The HRS is a longitudinal study of Americans over age 50, conducted every two years from 1992 to 2012; it collects information on economic, health, social, and other factors relevant to aging and retirement. DNA samples were collected in 2006 and 2008. Out of the collected samples, 13,129 individuals were put into genotyping production and 12,507 passed the University of Washington Genetics Coordinating Center’s standardized quality control process.

The HRS analysis was performed on 12,237 unrelated individuals and the 11,925 SNPs on Chromosome 16 that are common to those SNPs used in the FHS analysis of Section “The Framingham heart study”.

The estimation results are shown in the right panel of Table 3 and Table 4. We first note that the HBM estimates of the proportion of association SNPs are very close in the two studies: 0.34*%* and 0.42*%* for FHS and HRS respectively. Both data sets identified the same set of six genes for BMI including the well-known *FTO* gene. These genes account for about 25*%* of the genes identified in our analysis.

Forty SNPs are identified to be associated with BMI by the HBM using HRS data set, which are listed in the bottom panel of Table 6. Between the two studies, the HBM identifies three common SNPs to be associated with BMI: rs4782578, rs4784621 and rs9939606 (shown in red), as well as a few common genes (shown in blue). Furthermore, SNP rs9940128 identified using the HRS data is also on the *FTO* gene, and has been found before to be correlated with BMI by [16, 17] and [18].

## Conclusion

In this paper, we propose a Hierarchical Bayesian Model (HBM) that extends the MLM of [3]. Our model allows SNP effects on phenotypes of interest to follow a mixture distribution of a point mass at zero and a normal distribution. Our approach addresses the challenge of high-dimensionality in GWAS data by incorporating simultaneous selection of genetics variables that are jointly significant in predicting the phenotype. We employ several computing tricks that enable us to analyze a large number of SNPs (in the order of 100,000).

We demonstrate the applicability of our approach using both simulated and real data. The simulations are first used to show the accuracy and robustness of the estimation algorithm. We then analyze real data from the FHS and the HRS to identify SNPs on Chromosome 16 that are associated with the body mass index (BMI). The identified SNPs are consistent with earlier findings in the literature, and the results can be replicated across the two studies. The results from both the simulations and the real applications suggest that the MLM tends to over-estimate the proportion of total genetic variance over total phenotypic variance, i.e. PVE. The reason is that the MLM assumes that all the SNPs have effect on the phenotype, including those SNPs with small or noisy effects.

Our work offers a flexible framework that can be extended in several aspects. We now offer some discussion regarding potential future work directions. To analyze the whole-genome data, we can follow [19] and [20] to analyze each chromosome separately. We believe that more work is needed to rigorously study how to aggregate the results, and leave that for future work. The current assumption on the mixture distribution, i.e. a point mass at zero plus a normal distribution, may not be flexible enough to capture genetic effects in certain situations. We intend to relax the distributional assumption to a mixture of a point mass at zero plus a nonparametric distribution as in [21]. One challenge is that the computational short cut we used in this study for Gibbs Sampling might not remain effective for more flexible distributions; hence alternative algorithm have to be considered. Another direction of extension is to relax the independence assumption to allow potential dependence among SNPs within LD blocks. One difficulty then is the estimation of (potentially arbitrary) correlation structure among the SNPs. We are experimenting with adapting the principal factor approximation idea of [22] into our current framework.

## Methods

All research involving human subjects, human material, and human data in this paper has been performed in accordance with the Declaration of Helsinki, and with approval from the University of North Carolina-Chapel Hill Institutional Review Board.

The statistical setup of our model is closely related to that of [7, 23], and [24]. Our estimation algorithm combines the good features of the three methods, and is the fastest to compute, which is crucial for analyzing GWAS data [7]. First proposed a stochastic search algorithm in order to identify the subset of “promising” subsets predictors through multiple regression. The key feature of the study assumes that the slope of each regressor comes from a mixture of two normal distributions with different variances. The set of slopes with the smaller variance can be considered as being equal to 0. By employing a mixture normal distribution, [7] avoided discontinuity of the mixture between point mass and a normal distribution. However, each step of the iteration would involve all the regressors which is time-consuming, especially when the number of the regressors is large. In another closely related study, [23] explicitly considered the situation in which the regressors’ slopes are distributed as zero plus a continuous distribution [23], also allowed sign constraints on the continuous part of the distribution. Although Geweke’s approach incorporates more realistic assumptions compared with [7], one shortcoming is its slow computation, which makes it unrealistic for large scale genetic studies [24], also tackled the problem of gene selection using the Bayesian variable selection framework. Their algorithm is similar to ours in that both use computational shortcuts to derive the posterior distribution of the random effects conditional on the significant ones in each iteration. However, the proportion of the significant random effects *p* is pre-specified in their research, while we can estimate it in the process. We relax the known *p* assumption in our analysis by assuming a prior distribution for *p* and estimate it using its posterior distribution.

Automatic relevance determination (ARD) is a popular Bayesian variable selection approach [25]. ARD assumes that each random regressor slope follows a normal distribution with mean 0 and a (potentially) distinct variance. The hyperparameters, i.e. the variances, are estimated through maximizing the marginal likelihood, and the variables with zero variance estimates are pruned from the model. The flexibility of the hyperparameters and the estimation algorithm make it difficult to apply ARD to GWAS with a large number of SNPs, which is our primary interest. Our HBM can be viewed as a special case of ARD with only two choices for the hyper parameters: 0 for those non-associative SNPs and  for those associative SNPs, and our model is estimated via Gibbs sampling instead of direct likelihood maximization. Similar to the setup in [7], we use a latent variable *I*_*j*_ such that when *I*_*j*_=1, the random effect of the *j*^*th*^ SNP, *b*_*j*_, follows  and when *I*_*j*_=0, *b*_*j*_=0. In addition, *I*_*j*_ follows a Bernoulli distribution with Pr(*I*_*j*_=1)=*p*, the mixture probability. We seek to estimate the parameters, *p*, *β*,  and  in (1) and (2), as well as predict the random effects *b*.

Because of the large number of random effects, which equals the number of SNPs in this study, a faster algorithm is employed in our approach based on (1) and (2). The algorithm first modifies the prior distribution of the random effects *b* to the following mixture normal distribution:
6

When *σ* is a really small number (e.g. *σ*=0.01), the above mixture normal distribution is approximately a mixture distribution of a normal distribution plus a point mass at zero. Secondly, rather than drawing from the posterior distribution of all the random effects *b* as a vector, we modify the algorithm to draw *b*_*j*_ component wise conditional on the indicator *I*_*j*_. Specifically, if *I*_*j*_=1, *b*_*j*_ is drawn from the marginal conditional distribution *f*(*b*_*j*_|*I*_*j*_=1), and for *I*_*j*_=0, *b*_*j*_ is set to zero in each iteration. Thus in each iteration of the Gibbs sampling, the conditional distribution, *f*(*b*_*j*_|*I*_*j*_=1), would only involve the columns of **W** that correspond to *I*_*j*_=1. In practice, this algorithm speeds up the computation considerably especially in the case when the random effects *b* have a high dimension and the true mixture probability *p* is small. For example, it takes 21,727 and 2,004 seconds respectively using the stochastic search algorithm of [7] and our algorithm on a simulated data set with 5,000 SNPs and 5,000 individuals and the true mixture probability *p* as 0.1.

To complete the hierarchical model, we make the following prior assumptions: *p*∼*Beta*(1,1);  and  where *a*_1_=*b*_1_=*a*_2_=*b*_2_=0.001;  where *σ*_*a*_=10^5^.

The Gibbs sampling algorithm for estimating the HBM is provided in Table 1. After a burn-in period of 5,000 iterations, the MCMC samples , *t*=5,000,…,7,000, are obtained. Statistical inference and prediction can then be made based on the posterior distribution of these parameters.

## Availability of supporting data

Our study used phenotype and genotype data from two national studies in the United States: the Framingham Heart Study (FHS) and the Health Retirement Survey (HRS), both were conducted with support from NIH. Our group applied for, received approval for using, and downloaded the data from the National Center for Biotechnology Information Genotypes and Phenotypes Database (NCBI dbGaP). The FHS and HRS data should be available to researchers who follow the same application procedures.
